# Combining population-based administrative health records and electronic medical records for disease surveillance

**DOI:** 10.1186/s12911-019-0845-5

**Published:** 2019-07-02

**Authors:** Saeed Al-Azazi, Alexander Singer, Rasheda Rabbani, Lisa M. Lix

**Affiliations:** 1 Department of Community Health Sciences, University of Manitoba, S113-750 Bannatyne Avenue, Winnipeg, MB R3E 0W3 Canada; 2 George & Fay Yee Centre for Healthcare Innovation, University of Manitoba, Winnipeg, MB Canada; 3 Department of Family Medicine, University of Manitoba, Winnipeg, MB Canada

**Keywords:** Administrative data, Electronic medical records, Misclassification bias, Prevalence, Statistical model

## Abstract

**Background:**

Administrative health records (AHRs) and electronic medical records (EMRs) are two key sources of population-based data for disease surveillance, but misclassification errors in the data can bias disease estimates. Methods that combine information from error-prone data sources can build on the strengths of AHRs and EMRs. We compared bias and error for four data-combining methods and applied them to estimate hypertension prevalence.

**Methods:**

Our study included rule-based OR and AND methods that identify disease cases from either or both data sources, respectively, rule-based sensitivity-specificity adjusted (RSSA) method that corrects for inaccuracies using a deterministic rule, and probabilistic-based sensitivity-specificity adjusted (PSSA) method that corrects for error using a statistical model. Computer simulation was used to estimate relative bias (RB) and mean square error (MSE) under varying conditions of population disease prevalence, correlation amongst data sources, and amount of misclassification error. AHRs and EMRs for Manitoba, Canada were used to estimate hypertension prevalence using validated case definitions and multiple disease markers.

**Results:**

The OR method had the lowest RB and MSE when population disease prevalence was 10%, and the RSSA method had the lowest RB and MSE when population prevalence increased to 20%. As the correlation between data sources increased, the OR method resulted in the lowest RB and MSE. Estimates of hypertension prevalence for AHRs and EMRs alone were 30.9% (95% CI: 30.6–31.2) and 24.9% (95% CI: 24.6–25.2), respectively. The estimates were 21.4% (95% CI: 21.1–21.7), for the AND method, 34.4% (95% CI: 34.1–34.8) for the OR method, 32.2% (95% CI: 31.8–32.6) for the RSSA method, and ranged from 34.3% (95% CI: 34.1–34.5) to 35.9% (95% CI, 35.7–36.1) for the PSSA method, depending on the statistical model.

**Conclusions:**

The OR and AND methods are influenced by correlation amongst the data sources, while the RSSA method is dependent on the accuracy of prior sensitivity and specificity estimates. The PSSA method performed well when population prevalence was high and average correlations amongst disease markers was low. This study will guide researchers to select a data-combining method that best suits their data characteristics.

**Electronic supplementary material:**

The online version of this article (10.1186/s12911-019-0845-5) contains supplementary material, which is available to authorized users.

## Background

Prevalence and incidence are essential measures for disease surveillance, to describe the burden of disease in a population and compare health status across populations and over time. Routinely-collected electronic health databases, such as administrative health records (AHRs), which are captured for healthcare system management and remuneration, are important sources for estimating disease prevalence and incidence because they provide information for the entire population and can therefore be used for surveillance of both common and rare conditions [1–5]. As well, they systematically capture information over time, which enables monitoring of trends. Electronic medical records (EMRs), digital versions of patient medical charts, are also increasingly being used for disease surveillance because they have many of the same advantages as AHRs and they also capture clinical information such as body mass index, smoking, and alcohol use [6–9].

However, both AHRs and EMRs are prone to misclassification errors [5, 9–13], including false negative cases in which individuals are incorrectly classified as not having a disease and false positive cases in which individuals are incorrectly classified as having a disease [14]. The magnitude and types of errors in each of these data sources may not be the same [15–17], therefore one source should not be routinely recommended over the other source for population-based disease surveillance.

Combining information from EMRs and AHRs is an alternative to using one error-prone source over the other; data-combining methods capitalize on the strengths of each source for ascertaining cases to estimate chronic disease incidence and prevalence, and therefore help to reduce the impact of error. Data-combining methods based on both deterministic (i.e., rule-based) approaches and probabilistic models have been proposed [18–27]. However, there have been few comparisons of these methods [28–30]. Moreover, there have been limited investigations about the factors that may influence the accuracy of these methods.

The purpose of this study was to compare several methods for combining information from two error-prone data sources for estimating disease prevalence, including rule-based and model-based methods. The objectives were to: (1) compare the bias and precision of data-combining methods and (2) estimate hypertension prevalence from AHRs and EMRs alone as well as from four data-combining methods. We selected hypertension because it is a common measure of health status included in national and international disease surveillance reports [4, 31].

## Methods

The first objective relied on computer simulation techniques. The second objective was achieved using population-based AHR and EMR data from the province of Manitoba, Canada.

### Computer simulation

The computer simulation generated data from two sources using a model in which multiple disease markers are associated with the probability of disease presence/absence [32]. Specifically, we used copulas to generate multiple binary disease markers [33] for each data source. Copulas are constructed by specifying the joint distribution of correlated random variables that follow a standardized uniform distribution. The disease markers were assumed to be error-free with complete information. True disease status for each member of the population was generated from a Bernoulli distribution via a logistic regression model. To obtain the specified prevalence estimates, values of the regression coefficients and marker prevalence were selected based on previous epidemiological studies about hypertension [34, 35].

Subsequently, error-prone measures of disease status were generated based on pre-selected values of sensitivity (${Sn}_{Y_j}$) and specificity (${Sp}_{Y_j}$) for the *j*th data source (*j* = 1, 2) [36]. A conditional Bernoulli process was used [37]:

1 $$ {Y}_1=\mathrm{P}\left(D=1\right)\ \left[U<\mathrm{P}\left({Y}_1=1|\ D=1\right)\right]+\mathrm{P}\left(D=0\right)\ \left[U<1-\mathrm{P}\left({Y}_1=0|\ D=0\right)\right] $$

where *Y*_1_ is an error-prone measure of disease status from the first data source, P(*D* = 1) is the indicator of population disease status, P(*Y*_1_ *=* 1 | *D* = 1) and P(*Y*_1_ *=* 0 | *D* = 0) are the sensitivity and specificity for the first data source, and *U* is a random variable that follows a uniform distribution.

A total of 500 replications of the simulation model were produced for each of 144 combinations of simulation conditions; the four data-combining methods were applied to the data for each replication to estimate prevalence. The simulation conditions included all possible combinations of true population prevalence (*prev*_T_) of 10 and 20%, prevalence for each error-prone data source ($$ {prev}_{Y_1}, $$ $$ {prev}_{Y_2} $$) ranging in values from 5 to 18%, correlation between data sources ($$ {\rho}_{Y_1{Y}_2} $$) of 0.65 and 0.85, number of disease markers (*N*_*x*_) of 8 and 16, average correlation amongst the disease markers ($$ {\overline{\rho}}_x $$) of 0.00, 0.20, and 0.50 and correlation pattern amongst the disease markers ($$ {\overline{\rho}}_{x\ \left(\mathrm{pattern}\right)} $$) that was unstructured or exchangeable. True prevalence of 20% was chosen to reflect the estimated prevalence of hypertension observed in previous studies about population prevalence [38], whereas the true prevalence of 10% was chosen to reflect the lower prevalence observed in a specific sub-group like younger adults [39]. We focused on prevalence values for the data sources that were lower than the true population prevalence since both AHRs and EMRs often underestimate chronic disease cases [9–13]. Data source correlation values were chosen to test the effect of moderate and high associations between data sources [40]. The average correlation and correlation pattern were relevant for investigations about the PSSA method [20]. The data-combining methods were evaluated using percent absolute relative bias (RB) and mean square error (MSE) [41]. Percent absolute RB was calculated as:

2 $$ \mathrm{RB}=\kern0.5em \frac{\left|{prev}_{\mathrm{T}}-\overline{prev_{\mathrm{m}}}\right|}{prev_{\mathrm{T}}}\kern0.5em \times \kern0.5em 100 $$

where $\overline{prev_{\mathrm{m}}}$ is the mean prevalence for a data-combining method across the replications. MSE was calculated as $\mathrm{MSE}={\sigma^2}_{prev_{\mathrm{m}}}\kern0.5em +\kern0.5em {\left|\kern0.5em {prev}_{\mathrm{T}}\kern0.5em -\kern0.5em \overline{prev_{\mathrm{m}}}\kern0.5em \right|}^2,\kern0.5em \mathrm{where}\kern0.5em {\sigma^2}_{prev_{\mathrm{m}}}$is the variance of the estimates. The simulation study was conducted using R software version R-3.4.4 for Windows [42].

### Population-based data sources and study cohort

The study data for Objective 2 were AHRs and EMRs from the Manitoba Population Research Data Repository housed at the Manitoba Centre for Health Policy (MCHP), a research unit at the University of Manitoba. The province of Manitoba has universal healthcare, which means that virtually all health system contacts are captured in AHRs for the entire population of 1.3 million residents. The study observation period was fiscal years 2005/06 to 2008/09 (a fiscal year extends from April 1 to March 31).

AHRs included hospital discharge abstracts, physician billing claims, and Drug Program Information Network (DPIN) records. Hospital discharge abstracts contain records of discharges from acute care facilities; each abstract captures up to 25 diagnosis codes that use the World Health Organization’s International Classification of Diseases (ICD), 10th revision, Canadian version (ICD-10-CA). Physician billing claims are submitted by fee-for-service physicians to the ministry of health for provider remuneration. Each claim includes a single three-digit ICD-9-CM code for the diagnosis best reflecting the reason for the visit. The DPIN is an electronic, online, point-of-sale database that contains information about prescriptions filled by community pharmacies. Each approved drug is assigned a Drug Identification Number (DIN) by Health Canada; DINs can be linked to the World Health Organization’s Anatomical Therapeutic Chemical (ATC) codes [43].

EMRs used were obtained from the Manitoba Primary Care Research Network (MaPCReN) which is a practice-based research network comprised of consenting primary care providers (mostly family physicians). The MaPCReN repository includes information on health problems, billing data, medications, laboratory results, selected risk factors, referrals, and procedures for primary care patients [10]. EMRs from Manitoba has been previously used to evaluate the quality of these data for measuring hypertension [44]. Approximately 22% of the provincial population is represented in the MaPCReN repository, which covers all geographic regions and various practice configurations within the province [45].

EMRs and AHRs were linked using an encrypted unique personal health identification number (PHIN) available in the population registry; the registry captures information on dates of healthcare coverage, demographic characteristics, and location of residence.

The PHIN is available on each record in all of the data sources. Any identifying data, such as names and addresses were removed from the data by the provincial ministry of health prior to record linkage. Before linkage, key variables including sex, birth date, postal code, and PHIN were formatted in the same way on each file to account for formatting differences, such as capitalization, justification, and leading zeroes.

Validated case ascertainment algorithms for hypertension were applied to each data source [9, 12]. Table 1 lists the components of these algorithms, including ICD diagnosis codes and ATC prescription drug codes.

**Table 1:** Hypertension case ascertainment algorithms from administrative health records (AHRs) and electronic medical records (EMRs)

Data source	Contact frequency, source and duration	ICD 9-CM/10-CA diagnosis codes	ATC medication codes
AHR	1 + H or 2 + P in 2 years	ICD-9-CM: 401–405 ICD-10-CA: I10-I13, I15	
EMR	(2 + P in 2 years) or 1 + PL or 1 + Rx ever	ICD-9-CM: 401–405	C07AB04, C09XA02, C03DB01, C08CA01, C07AB03, C07CB03, C09AA07, C09AA01, C07AG02, C03BA04, C09AA08, C09AA02, C09BA02, C09CA02, C09DA02, C08CA02, C09AA09,C03AA03, C03EA01, C03BA11, C09CA04, C09DA04, C09AA03, C09BA03, C09DA01, C02LB01, C03BA08, C09CA07, C07AA06, C09AA10, C03DB02, C09CA03, C08DA01

*H* Hospital discharge abstract, *P* Physician billing claim, *PL* Problem list, *Rx* Drug codes; *ICD-9-CM/10-CA* International Classification of Diseases, 9th Revision, Clinical Modification and 10th version of the Canadian version, *ATC* Anatomic Therapeutic Chemical classification system

The study cohort included Manitoba residents 18+ years of age with at least one encounter in EMR data during the study observation period. The EMR data were linked to AHR data for all cohort members. To be retained in the cohort, an individual required a minimum of 7 years of health insurance coverage before the study index date and 7 years of coverage after the study index date, in order to implement the EMR case ascertainment algorithm for hypertension [46]. The study index date was the date of the individual’s first record in EMR data.

### Model covariates

Socio-demographic and comorbidity measures were used to describe the study cohort and as covariates (i.e., markers) in the statistical model for the probabilistic data-combining method. Socio-demographic measures, which included sex, age group (18–44, 45–64, 65+ years), income quintile, and region of residence, were defined at the study index date. Income quintile is an area-level measure of socioeconomic status defined using Statistics Canada Census data and based on total household income for dissemination areas, the smallest geographic unit for which Census data are publicly released [47]. Postal codes from the population registry were used to assign individuals to income quintiles. Region was based on regional boundaries and was defined as Winnipeg and non-Winnipeg.

Comorbidity measures included the Charlson comorbidity score (CCS) and multiple disease-specific measures. The CCS is a summary measure based on ICD diagnosis codes from hospital discharge abstracts and physician billing claims [48]; it was derived using data for the one-year period prior to the study index date. The CCS was defined as a categorical variable with values of 0, 1 to 2, and 3+. Disease-specific covariates included chronic obstructive pulmonary disease (COPD), diabetes, depression, dementia, obesity, cerebrovascular disease, congestive heart failure, coronary heart disease, renal disease, and substance abuse, all of which have been used in previous research as indicators of hypertension in probabilistic models [49–51]. The first five covariates were defined from both AHRs and EMRs. The remaining covariates were defined from AHRs only because EMR case ascertainment algorithms have not been developed. Case ascertainment algorithms for AHRs were based on the two-year period prior to the index date in accordance with previous recommendations [49], while EMR case ascertainment algorithms did not have a time period requirement. Finally, obesity, another covariate for the probabilistic model, was defined from EMRs (obese = body mass index > 30.0; not obese = body mass index ≤30.0; missing).

### Data-combining methods

Four data-combining were selected based on previous research [21]. We included rule-based OR and AND methods, which use a deterministic rule to classify individuals as having the target disease or not having the target disease. The OR method identified individuals as hypertension cases if they met the case ascertainment algorithm for either EMRs or AHRs, and the AND method identified individuals as hypertension cases if they met the case ascertainment algorithm for both EMRs and AHRs [24]. The OR and AND methods assume: (1) observed disease status is 100% sensitive and specific, and (2) observed disease status from two data sources is conditionally independent on the true disease status.

We also considered a rule-based sensitivity and specificity adjusted (RSSA) method, which uses information about the accuracy of case ascertainment algorithms from prior validation studies to correct the estimated number of true disease cases [25, 26, 52]. The number of individuals ascertained as disease cases was weighted by the average values of sensitivity and specificity for each source identified from three Canadian validation studies about hypertension [5, 9–13]. Specifically, the average sensitivity and specificity values used were 0.72 and 0.95 for AHRs and 0.87 and 0.90 for EMRs. The RSSA method assumes that observed disease status from the two data sources is conditionally independent.

The probabilistic sensitivity-specificity (PSSA) method was also considered; it assumes that true disease status is associated with disease markers [20]. The sensitivities and specificities of the two data sources are modelled via a Bayesian regression model with a probit link function. The model can be decomposed into an outcome model (i.e., true outcome given disease markers) and a reporting model (i.e., reported status given true outcome and disease markers). It was assumed that the joint distribution of the reported (i.e., observed) disease status was conditional on the true disease status and observed markers. Using a Gibbs sampling technique, values of the unobserved true disease status is sampled from the posterior distribution conditional on the disease markers [53]. Model convergence was assessed using diagnostics recommended in previous research [54].

We considered four models for the PSSA method using different subsets of covariates (i.e., markers) based on theory, previous research, and empirical estimates of correlation amongst the covariates. For Model 1, which was the full model, the covariates included all socio-demographic variables, the CCS, and all disease-specific markers. For Model 2, only EMR-defined measures of COPD, diabetes, dementia, depression and obesity were selected for model inclusion. In addition, given that the CCS includes some comorbid conditions already identified as disease-specific markers, it was excluded. For Model 3, we excluded markers with correlations > |0.60|. For Model 4, which was the reduced model, we limited our attention to covariates strongly associated with hypertension prevalence based on previous research [50, 51], including age, sex, diabetes, obesity, cardiovascular disease, COPD (a proxy for smoking status) [55] and substance use.

For each of the PSSA models, visual graphical assessment using traceplots demonstrated that model convergence was reached after the 500th iteration [see Additional file 1]. We ran a total of 10,000 iterations of the Gibbs sampler for each model. In addition, we used Gelman–Rubin diagnostics to ensure the Potential Scale Reduction Factor (PSRF) of all parameters was close to one [56], suggesting that 10,000 iterations were sufficient for attaining convergence. Once we decided that the chain has converged at iteration 500, we discarded the first 500 samples as burn-in samples and used the remaining 9500 samples for inference.

### Statistical analysis for numeric example

Descriptive analyses were conducted using frequencies and percentages. Associations amongst the covariates and case ascertainment algorithms were estimated using tetrachoric and polychoric correlations [57].

Hypertension prevalence estimates and 95% confidence intervals (95% CIs) were calculated for each data combining-method and for each data source on its own. We also calculated sex and age-group stratified estimates and their 95% CIs. For the OR and AND methods, we assumed a normal approximation to the binomial distribution when calculating the 95% CIs. For the RSSA and PSSA methods we constructed 95% CIs using the percentile bootstrap method; the number of bootstrap samples was set to 999 following previous recommendations [58].

Model fit was assessed for the PSSA method using the Deviance Information Criterion (DIC) [59], which is a penalized measure of the log of the likelihood function. Smaller values of the DIC indicate a better fitting model [60].

## Results

### Computer simulation

The simulation results are reported in Table 2; for the PSSA method we reported results for an exchangeable correlation amongst the model covariates; similar results were obtained for an unstructured correlation and are therefore not reported. Absolute RB ranged from 0.2 to 108.8% and MSE ranged from 0.00 to 6.16 across the simulation conditions.

**Table 2:** Percent absolute relative bias (RB) and mean squared error (MSE) for computer simulation study

$$ {prev}_{Y_1}, $$ $$ {prev}_{Y_2} $$	$$ {\overline{\rho}}_x $$	**RB;** ***prev***_**T**_ **= 20%**									
		${\boldsymbol{\rho}}_{{\boldsymbol{Y}}_{\mathbf{1}}{\boldsymbol{Y}}_{\mathbf{2}}}$ **= 0.85**					${\boldsymbol{\rho}}_{{\boldsymbol{Y}}_{\mathbf{1}}{\boldsymbol{Y}}_{\mathbf{2}}}$ **= 0.65**				
		**OR**	**AND**	**RSSA**	**PSSA (16)**	**PSSA (8)**	**OR**	**AND**	**RSSA**	**PSSA (16)**	**PSSA (8)**
**18, 15%**	0.00	9.5	47.5	7.5	9.5	11.3	23.1	59.4	1.3	48.3	49.5
	**0.20**	**9.0**	**47.7**	**7.9**	**2.1**	**7.4**	22.9	59.4	1.5	41.7	54.3
	0.50	10.1	47.2	7.0	24.3	21.1	23.7	59.1	0.9	99.0	78.6
18, 10%	0.00	0.3	58.6	18.2	1.1	3.0	10.8	67.1	12.8	28.9	37.5
	0.20	0.9	58.9	18.7	5.9	5.9	10.5	67.2	13.0	31.1	54.3
	0.50	0.2	58.3	17.7	48.8	26.1	11.2	66.8	12.4	108.8	90.1
15, 15%	0.00	4.1	49.2	11.5	3.7	4.3	17.6	61.7	5.8	41.3	42.5
	0.20	3.6	49.4	12.0	3.1	3.6	17.2	61.9	6.1	37.6	50.1
	0.50	4.8	48.7	10.9	20.5	12.5	18.1	61.5	5.3	102.0	70.7
$$ {prev}_{Y_1}, $$ $$ {prev}_{Y_2} $$	$$ {\overline{\rho}}_x $$	**MSE;** ***prev***_**T**_ **= 20%**									
		${\boldsymbol{\rho}}_{{\boldsymbol{Y}}_{\mathbf{1}}{\boldsymbol{Y}}_{\mathbf{2}}}$ **= 0.85**					${\boldsymbol{\rho}}_{{\boldsymbol{Y}}_{\mathbf{1}}{\boldsymbol{Y}}_{\mathbf{2}}}$ **= 0.65**				
		**OR**	**AND**	**RSSA**	**PSSA (16)**	**PSSA (8)**	**OR**	**AND**	**RSSA**	**PSSA (16)**	**PSSA (8)**
**18, 15%**	0.00	0.04	0.90	0.02	0.06	0.47	0.22	1.41	< 0.01	0.99	1.76
	**0.20**	**0.03**	**0.91**	**0.03**	**0.02**	**0.52**	0.21	1.41	< 0.01	0.82	2.25
	0.50	0.04	0.89	0.02	1.06	1.47	0.23	1.40	0.00	4.68	4.46
18, 10%	0.00	< 0.01	1.37	0.13	0.02	0.69	0.05	1.80	0.07	0.40	1.70
	0.20	< 0.01	1.39	0.14	0.06	1.16	0.05	1.80	0.07	0.70	3.48
	0.50	< 0.01	1.36	0.13	2.28	2.32	0.05	1.79	0.06	5.36	6.16
15, 15%	0.00	0.01	0.97	0.05	0.03	0.46	0.13	1.53	0.01	0.74	1.62
	0.20	0.01	0.98	0.06	0.02	0.72	0.12	1.53	0.02	0.74	2.20
	0.50	0.01	0.95	0.05	1.03	1.29	0.13	1.51	0.01	4.84	3.96
$$ {prev}_{Y_1}, $$ $$ {prev}_{Y_2} $$	$$ {\overline{\rho}}_x $$	**RB;** ***prev***_**T**_ **= 10%**									
		${\boldsymbol{\rho}}_{{\boldsymbol{Y}}_{\mathbf{1}}{\boldsymbol{Y}}_{\mathbf{2}}}$ **= 0.85**					${\boldsymbol{\rho}}_{{\boldsymbol{Y}}_{\mathbf{1}}{\boldsymbol{Y}}_{\mathbf{2}}}$ **= 0.65**				
		**OR**	**AND**	**RSSA**	**PSSA (16)**	**PSSA (8)**	**OR**	**AND**	**RSSA**	**PSSA (16)**	**PSSA (8)**
8, 7%	0.00	11.5	55.8	8.4	9.7	35.0	29.6	67.1	1.1	76.2	154.9
	0.20	10.5	56.1	9.2	42.6	37.8	28.7	67.4	0.3	196.7	217.1
	0.50	13.1	54.9	7.1	216.1	43.3	30.7	66.6	2.0	307.6	286.5
8, 5%	0.00	2.9	59.7	16.0	1.1	50.5	12.2	73.4	13.5	45.3	114.9
	0.20	3.2	59.7	15.8	10.7	85.1	12.0	73.9	13.8	235.2	273.4
	0.50	3.8	59.2	15.3	230.5	198.2	14.2	73.3	12.1	322.2	334.8
5, 5%	0.00	14.7	70.0	30.9	6.3	92.1	7.4	78.7	28.4	61.0	193.0
	0.20	15.4	70.2	31.5	134.4	149.1	8.0	78.7	28.8	271.0	217.9
	0.50	13.6	69.5	30.0	275.7	222.1	6.3	78.2	27.4	333.8	375.0
$$ {prev}_{Y_1}, $$ $$ {prev}_{Y_2} $$	$$ {\overline{\rho}}_x $$	**MSE;** ***prev***_**T**_ **= 10%**									
		${\boldsymbol{\rho}}_{{\boldsymbol{Y}}_{\mathbf{1}}{\boldsymbol{Y}}_{\mathbf{2}}}$ **= 0.85**					${\boldsymbol{\rho}}_{{\boldsymbol{Y}}_{\mathbf{1}}{\boldsymbol{Y}}_{\mathbf{2}}}$ **= 0.65**				
		**OR**	**AND**	**RSSA**	**PSSA (16)**	**PSSA (8)**	**OR**	**AND**	**RSSA**	**PSSA (16)**	**PSSA (8)**
8, 7%	0.00	0.01	0.31	0.01	0.28	2.01	0.09	0.45	< 0.01	0.87	6.33
	0.20	0.01	0.31	0.01	1.29	1.31	0.08	0.45	< 0.01	5.19	8.53
	0.50	0.02	0.30	0.01	5.60	6.72	0.10	0.44	< 0.01	9.97	12.77
8, 5%	0.00	< 0.01	0.36	0.03	0.12	2.78	0.02	0.54	0.02	0.28	4.31
	0.20	< 0.01	0.36	0.03	0.59	3.59	0.02	0.55	0.02	7.28	12.63
	0.50	< 0.01	0.35	0.02	6.39	8.45	0.02	0.54	0.02	10.91	16.73
5, 5%	0.00	0.02	0.49	0.10	0.57	6.96	0.01	0.62	0.08	1.92	9.37
	0.20	0.02	0.49	0.10	4.82	8.11	0.01	0.62	0.08	9.11	9.70
	0.50	0.02	0.48	0.09	8.71	10.08	< 0.01	0.61	0.08	11.79	18.41

*OR* Rule-based OR method, *AND* Rule-based AND method, *RSSA* Rule-based sensitivity-specificity adjusted method, *PSSA* Probabilistic-based sensitivity-specificity adjusted; *prev*_T_ denotes true population prevalence; $$ {prev}_{Y_1}, $$ $$ {prev}_{Y_2} $$ denotes outcome prevalence; $$ {\rho}_{Y_1{Y}_2} $$ denotes correlation between data sources; $$ {\overline{\rho}}_x $$ denotes average correlation amongst disease markers using the exchangeable correlation pattern. * in PSSA(*) denotes the number of model markers (i.e., covariates) for PSSA method; we multiplied each MSE value by 100; The bolded simulation condition are consistent with the conditions observed for our numeric example of hypertension

When true prevalence was 20%, the outcome prevalence combination of (18, 10%) for the two data sources resulted in the smallest percent absolute RB and MSE values for the OR method. However, for the AND, RSSA and PSSA methods, the absolute RB and MSE values were smallest for outcome prevalence combination (18, 15%). The RSSA method had the smallest absolute RB when ${\rho}_{y_1{y}_2}$ = 0.65 and the OR method resulted in average absolute RB that was the smallest when ${\rho}_{y_1{y}_2}$ = 0.85.

When the average marker correlation was either ${\overline{\rho}}_x$ = 0.00 or ${\overline{\rho}}_x$ = 0.20 and true prevalence was 20%, the PSSA method had the smallest absolute RB (3.7%) when ${\rho}_{y_1{y}_2}$ = 0.85 and outcome prevalence was (15, 15%). As the average marker correlation increased from ${\overline{\rho}}_x$ = 0.00 to ${\overline{\rho}}_x$ = 0.50, the absolute RB and MSE values for the PSSA method increased by more than 90%, irrespective of the correlation between the data sources. The absolute RB showed very little variation (less than 7%) when the average marker correlation was ${\overline{\rho}}_x$ = 0.00 compared to ${\overline{\rho}}_x$ = 0.20. When the average marker correlation was zero (i.e., independent markers), the PSSA method produced prevalence estimates that were stable. This result suggests that each of the markers was providing unique information to the model.

When true prevalence was 10%, Table 2 revealed that absolute RB ranged from 0.3 to 375.0% and MSE ranged from < 0.01 to 18.41 across the simulation conditions. The RSSA method had the smallest percent absolute RB and MSE when the outcome prevalence combination was (8, 7%), regardless of the correlation between data sources. As outcome prevalence went from (8, 7%) to (5, 5%), performance of the RSSA and AND methods got worse. For example, the percent absolute RB and MSE for the RSSA method went from 8.4% and 0.01 to 30.9% and 0.10, when ${\rho}_{y_1{y}_2}$ = 0.85. On the other hand, when ${\rho}_{y_1{y}_2}$ = 0.65, the average absolute RB and MSE went from 1.1% and < 0.01 to 28.8% and 0.08. The OR method resulted in absolute RB that was the smallest when the outcome prevalence was (8, 5%) and (5, 5%) regardless of the correlation between the data sources. For example, for outcome (8, 5%), the average absolute RB and MSE were 3.3% and < 0.01 when ${\rho}_{y_1{y}_2}$ = 0.85, and 12.8% and 0.02 when ${\rho}_{y_1{y}_2}$ = 0.65.

The PSSA method had the smallest absolute RB (1.1 and 6.3%) for outcome prevalence (8, 5%) and (5, 5%) when ${\overline{\rho}}_x$ = 0.00 and correlation between the data sources was ${\rho}_{y_1{y}_2}$ = 0.85. As the average marker correlation increased, the absolute RB and MSE values of the PSSA method increased substantially. For example, under outcome prevalence (8, 5%) and ${\rho}_{y_1{y}_2}$ = 0.85, the PSSA method had absolute RB of 1.1, 10.7 and 230.5% when the average marker correlation was 0.00, 0.20 and 0.50, respectively. When *N*_*x*_ = 8, the values of MSE for the PSSA method increased. For example, under outcome prevalence (8, 5%) and ${\overline{\rho}}_x$ = 0.00, the MSE value went from 0.12 to 2.78 when ${\rho}_{y_1{y}_2}$ = 0.85 and 0.28 to 4.31 when ${\rho}_{y_1{y}_2}$ = 0.65. Under all of the three outcome prevalence conditions, average absolute RB and MSE values of the PSSA method increased as the average marker correlation increased. As the correlation between the data sources went from ${\rho}_{y_1{y}_2}$ = 0.85 to ${\rho}_{y_1{y}_2}$ = 0.65, average absolute RB and MSE values increased substantially. For example, under outcome prevalence (8, 7%) and ${\rho}_{y_1{y}_2}$ = 0.85, the absolute RB values were 35.0, 37.8 and 43.3% when ${\overline{\rho}}_x$ = 0.00, 0.20 and 0.50, and 154.9, 217.1 and 286.5% when ${\rho}_{y_1{y}_2}$ = 0.65.

The results showed an increase in the absolute RB and MSE for each data-combining method when true prevalence was 10% compared with when it was 20%. In terms of the effect of the correlation between data sources, the absolute RB and MSE for the OR, AND and PSSA methods became smaller as the correlation increased from ${\rho}_{y_1{y}_2}$ = 0.65 to ${\rho}_{y_1{y}_2}$ = 0.85. The best results were obtained for the RSSA when ${\rho}_{y_1{y}_2}$ = 0.65 and the OR method when ${\rho}_{y_1{y}_2}$ = 0.85.

The effect of the average marker correlation on performance of the PSSA method was evident for all simulation conditions. The estimated prevalence became more biased as correlation increased. The percent absolute RB and MSE across all simulation conditions were 46.7% and 1.86 when ${\overline{\rho}}_x$ = 0.00 and 160.4% and 6.68 when ${\overline{\rho}}_x$ = 0.50.

### Results for numeric example

**Fig. 1:**
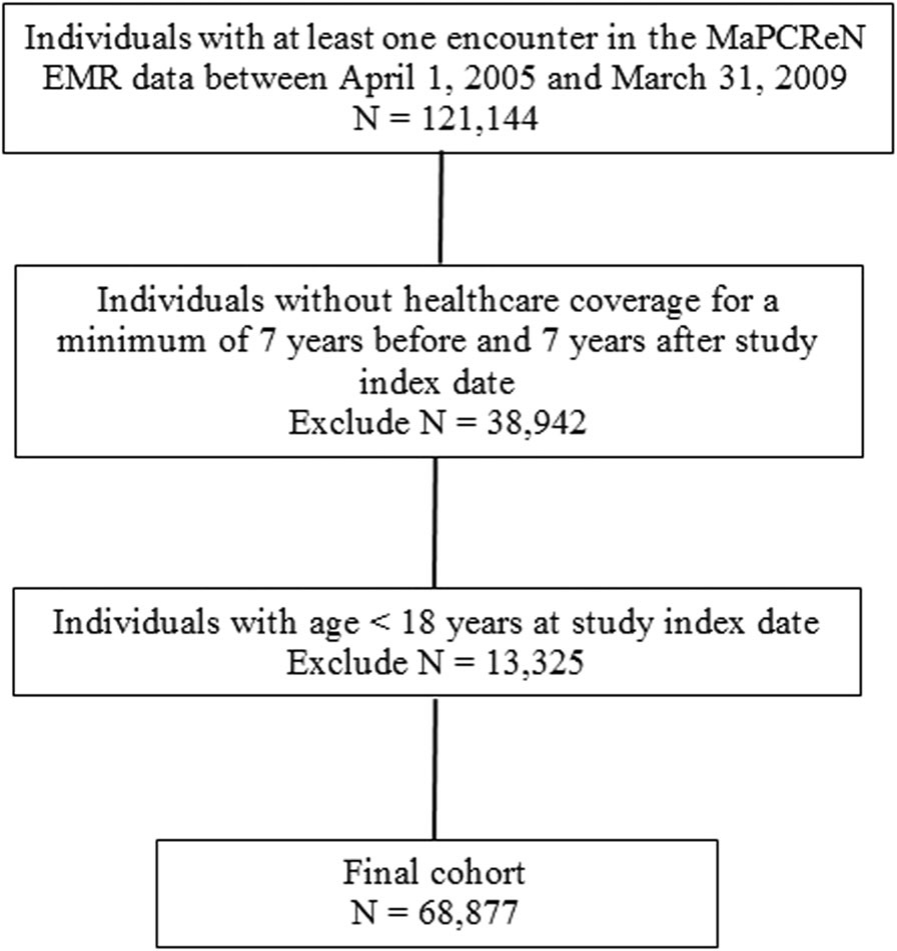
Study flowchart

A total of *N* = 121,144 individuals had at least one encounter in EMRs that could be linked to AHRs in the study observation period. After exclusions, the study cohort included *n* = 68,877 individuals (Fig. 1). Close to half of the individuals in the cohort were between 18 and 44 years of age. Slightly more than half of the cohort members were female and the majority were urban residents. Cohort members were equally distributed across most income quintiles, with the exception of the lowest quintile where they tended to be under-represented. More than 83% of the individuals in the cohort had a CCS score of 0 (Table 3).

**Table 3:** Socio-demographic characteristics and case ascertainment markers for the study cohort

Characteristics	Frequency	%
Sex		
Male	29,802	43.3
Female	39,075	56.7
Age group		
18–44 years	33,007	47.9
45–64 years	26,243	38.1
65+ years	9627	14.0
Region		
Non-Winnipeg	30,871	44.8
Winnipeg	38,006	55.2
Income quintile		
Not found	8888	12.9
Q1 (lowest)	8858	12.9
Q2	10,278	14.9
Q3	12,154	17.6
Q4	14,106	20.5
Q5 (highest)	14,593	21.2
Charlson Comorbidity Score		
0	57,649	83.7
1 to 2	10,348	15.0
3+	880	1.3
AHR-defined diseases		
Cerebrovascular disease	916	1.3
Congestive heart failure	558	0.8
COPD	1287	1.9
Coronary heart disease	2623	3.8
Dementia	625	0.9
Depression	7098	10.3
Diabetes	4176	6.1
Obesity	1623	2.4
Renal disease	916	1.3
Substance abuse	1387	2.0
EMR-defined diseases		
COPD	181	0.3
Dementia	1130	1.6
Depression	11,005	16.0
Diabetes	6435	9.3
Obesity	15,191	22.1

*Q* Income quintile, *COPD* Chronic obstructive pulmonary disease

In terms of the disease-specific covariates, individuals with diagnosed depression constituted 10.3% of the study cohort when identified from AHRs and 16.0% when identified from EMRs. A total of 1.9% of the study cohort had COPD when identified from AHRs and 0.3% when identified from EMRs.

The tetrachoric correlation for AHR and EMR case ascertainment algorithms was 0.90 (95% CI: 0.89–0.90). When stratifying the cohort by sex, the association between AHR and EMR case ascertainment algorithms was similar for males, with a value of 0.88 (95% CI: 0.88–0.90), and for females, with a value of 0.90 (95% CI: 0.90–0.91). Across age groups, the correlation coefficient had values of 0.89 (95% CI: 0.88–0.90) for ages 18 to 44 years, 0.87 (0.86–0.87) for ages 45 to 64 years, and 0.76 (95% CI: 0.74–0.77) for ages 65+ years.

**Fig. 2:**
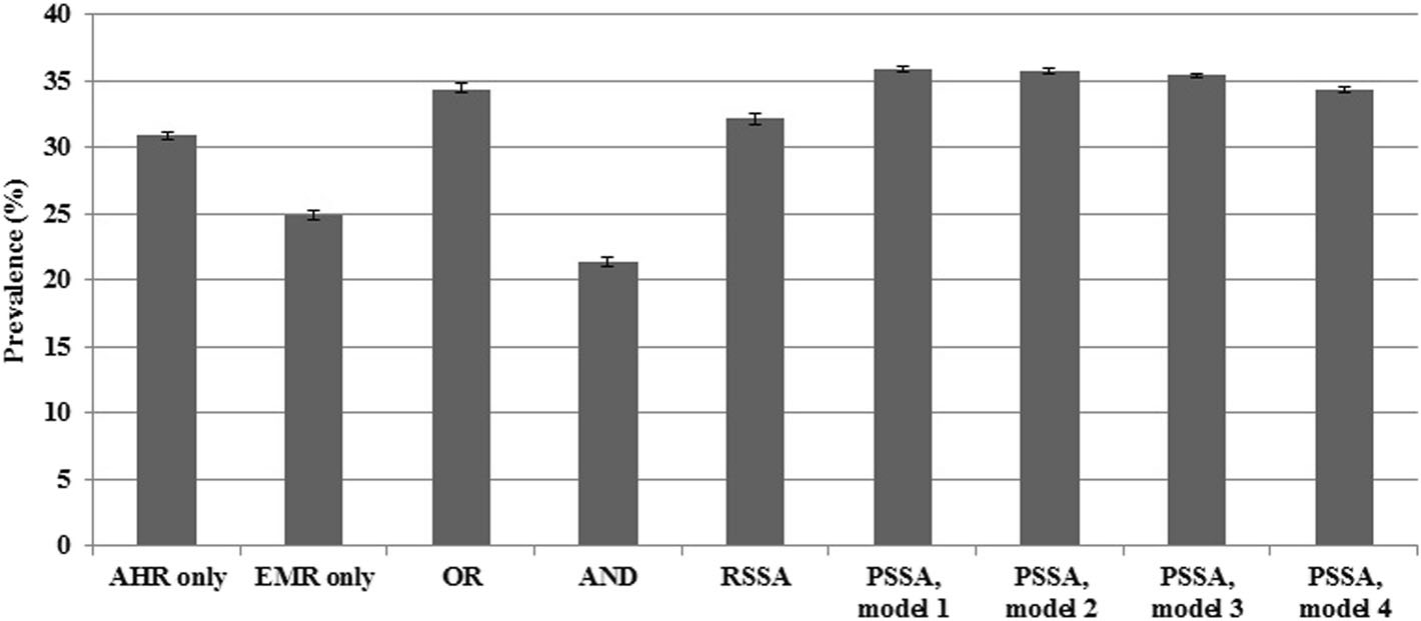
Hypertension prevalence estimates (%) for data-combining methods in the numeric example. Note: Error bars represent 95% confidence intervals; OR = rule-based OR method; AND = rule-based AND method; RSSA = rule-based sensitivity-specificity adjusted method; PSSA = probabilistic-based sensitivity-specificity adjusted method

The estimated hypertension prevalence using each data-combining method for the entire study cohort is shown in Fig. 2; the results stratified by sex and age group are reported in Table 4. The prevalence estimates for AHR and EMR case ascertainment algorithms had values of 30.9% (95% CI: 30.6–31.2) and 24.9% (95% CI: 24.6–25.2), respectively, which were significantly different. The estimated prevalence using the OR method was close to the estimate for AHRs (34.4%; 95% CI: 34.1–34.8). The AND method produced the lowest estimate. The RSSA method produced an estimate substantially lower than the OR method.

**Table 4:** Hypertension prevalence estimates (%) from administrative health records (AHRs) and electronic medical records (EMRs) in the numeric example

Data Source/Method	Males (95% CI)	Females (95% CI)	18–44 years (95% CI)	45–64 years (95% CI)	65+ years (95% CI)
AHR only	31.7 (31.2–32.2)	30.3 (29.8–30.8)	10.3 (10.0–10.6)	40.5 (39.9–41.1)	75.3 (74.4–76.2)
EMR only	26.0 (25.5–26.5)	24.1 (23.7–24.5)	9.0 (8.7–9.3)	33.5 (32.9–34.1)	56.4 (55.4–57.4)
OR	35.7 (35.2–36.2)	34.0 (33.5–34.5)	12.8 (12.4–13.2)	45.3 (44.7–45.9)	78.8 (78.0–79.6)
AND	22.1 (21.6–22.6)	20.9 (20.5–21.3)	6.4 (6.1–6.7)	28.7 (28.1–29.3)	53.0 (52.0–54.0)
RSSA	33.4 (32.8–33.9)	31.3 (30.6–31.8)	11.9 (11.6–12.3)	42.2 (41.6–42.8)	73.8 (72.9–74.7)
PSSA, model 1	37.1 (36.8–37.3)	34.9 (34.7–35.1)	13.9 (13.7–14.2)	46.9 (46.7–47.3)	79.7 (79.4–80.0)
PSSA, model 2	37.0 (36.8–37.2)	34.7 (34.5–35.0)	13.6 (13.4–13.9)	46.1 (45.9–46.4)	79.4 (79.1–79.7)
PSSA, model 3	36.5 (36.2–36.7)	34.5 (34.3–34.7)	12.8 (12.6–13.0)	46.3 (46.0–46.6)	79.4 (79.1–79.8)
PSSA, model 4	35.1 (34.9–35.4)	33.2 (32.9–33.5)	12.2 (11.9–12.4)	44.8 (44.5–45.1)	79.1 (78.8–79.5)

*CI* Confidence interval, *OR* Rule-based OR method, *AND* Rule-based AND method, *RSSA* Rule-based sensitivity-specificity adjusted method, *PSSA* Probabilistic-based sensitivity-specificity adjusted method, PSSA, model 1 covariates are sex, age group, region, income quintile, Charlson comorbidity score, chronic obstructive pulmonary disease (A, E), diabetes (A, E), depression (A, E), dementia (A, E), obesity (A, E), cerebrovascular disease (A), congestive heart failure (A), coronary heart disease (A), renal disease (A), substance abuse (A); PSSA, model 2 covariates are sex, age group, region, income quintile, chronic obstructive pulmonary disease (E), diabetes (E), depression (E), dementia (E), obesity (E), cerebrovascular disease (A), congestive heart failure (A), coronary heart disease (A), renal disease (A), substance abuse (A); PSSA, model 3 covariates are sex, age group, region, income quintile, chronic obstructive pulmonary disease (E), diabetes (E), depression (E), dementia (E), obesity (E), coronary heart disease (A), renal disease (A), substance abuse (A); PSSA, model 4 covariates are sex, age group, chronic obstructive pulmonary disease (E), diabetes (E), obesity (E), coronary heart disease (A), congestive heart failure (A), substance abuse (A); A and E denote disease-specific covariates that were identified from AHRs and EMRs, respectively

For the PSSA method, the mean absolute correlation values amongst the covariates included in Models 1 through 4 were: 0.18, 0.17, 0.13, and 0.16, respectively. Model 1 produced the highest prevalence estimate of 35.9% (95% CI: 35.7–36.1). Model 4 had the lowest estimate at 34.3% (95% CI: 34.1–34.5); these estimates were significantly different. Model 4 resulted in the lowest DIC (Table 5). As Table 4 reveals, similar patterns were observed for the data-combining methods across age groups as well as for males and females. The PSSA model fit statistics also produced consistent results, regardless of the stratification variables.

**Table 5:** Model fit statistics for the PSSA method in the numeric example

Model	Overall	Males	Females	18–44 years	45–64 years	65+ years
1	167,249	73,418	93,565	42,925	71,311	26,719
2	166,994	73,405	93,493	42,921	70,983	26,554
3	166,506	73,181	93,351	42,421	71,033	26,622
4	**165,719**	**72,706**	**92,688**	**42,220**	**70,483**	**26,525**

*PSSA* Probabilistic-based sensitivity-specificity adjusted method, PSSA, model 1 covariates are sex, age group, region, income quintile, Charlson comorbidity score, chronic obstructive pulmonary disease (A, E), diabetes (A, E), depression (A, E), dementia (A, E), obesity (A, E), cerebrovascular disease (A), congestive heart failure (A), coronary heart disease (A), renal disease (A), substance abuse (A); PSSA, model 2 covariates sex, age group, region, income quintile, chronic obstructive pulmonary disease (E), diabetes (E), depression (E), dementia (E), obesity (E), cerebrovascular disease (A), congestive heart failure (A), coronary heart disease (A), renal disease (A), substance abuse (A); PSSA, model 3 covariates are sex, age group, region, income quintile, chronic obstructive pulmonary disease (E), diabetes (E), depression (E), dementia (E), obesity (E), coronary heart disease (A), renal disease (A), substance abuse (A); PSSA, model 4 covariates are sex, age group, chronic obstructive pulmonary disease (E), diabetes (E), obesity (E), coronary heart disease (A), congestive heart failure (A), substance abuse (A); A and E denote disease-specific markers that were identified from AHRs and EMRs, respectively; Values in bold-face font represent the best-fitting model

## Discussion

Four data-combining methods that use information from two error-prone data sources for ascertaining chronic disease cases were compared. A simulation study was conducted to evaluate the performance of the methods. Then a numeric example for hypertension prevalence estimation was applied to real-world data. The investigated methods can benefit population health surveillance programs that inform health promotion and chronic disease prevention initiatives.

Under simulation conditions in which the two data sources were highly correlated, the estimated prevalence from the OR method was only slightly biased. For simulation conditions in which the two data sources were not highly correlated, the RSSA method had the lowest absolute RB and MSE among all other data-combining methods. Performance of the PSSA method was influenced by both the number of covariates and magnitude of their correlation.

In the numeric example, there was a high correlation between the AHR and EMR case ascertainment algorithms for hypertension, which provided a limited margin of improvement for the data-combining methods. The high degree of overlap left a small number of individuals classified as disease cases in one data source but not the other. Other studies have found a high degree of association between these two data sources for conditions with well-defined diagnostic criteria, including hypertension and diabetes [40, 61].

In our study cohort, the naïve estimates of hypertension prevalence from AHRs and EMRs were higher than those obtained from three Canadian studies, which had values of 19.6 and 21.3% for AHRs [13, 31, 39] and 22.8% for EMRs [46]. However, our results are consistent with those from another Canadian study that estimated hypertension prevalence to be between 27 and 30% using AHRs [5]. The patterns in terms of sex and age stratified prevalence estimates were consistent with previous studies [5, 39, 49], which lends face validity to our findings.

Amongst the rule-based methods, the AND and RSSA methods produced estimates of prevalence that were significantly lower than the OR method. This was somewhat surprising given the high degree of correlation between AHR and EMR case definitions. However, it also points to the need for almost complete overlap between the two data sources for the AND method to produce similar results to the OR method. Prevalence estimates for the PSSA method were similar for Models 1 through 3, but were significantly lower for Model 4 than for Model 1. The low variation in prevalence estimates for the first three models might be attributed to the low mean correlation amongst the markers. Our simulation study revealed that when the average correlation amongst the marker was zero (i.e., independent markers), the PSSA method produced prevalence estimates that were unbiased. The low correlation amongst the markers suggest that each marker was providing unique information to the model.

This study has some limitations. First, the simulation study focused on a limited number of simulation conditions. At the same time, we selected scenarios that are representative of real-world data [34, 35, 38]. Another limitation is that we focused on only a single chronic disease in our numeric example, and it had a relatively high prevalence. Greater differences across data-combining methods might be revealed for a chronic disease having lower prevalence in the population. We selected hypertension in part because a number of prior studies have demonstrated the feasibility of using administrative data for case ascertainment.

The key strength of this study was the use of both computer simulation and a real numeric example to investigate data-combining methods. We compared methods using two population-based data sources that are available in many jurisdictions worldwide. Moreover, this research investigated different sets of case ascertainment markers when applying the PSSA method, to assess the utility and feasibility of these markers as proxy measures of hypertension.

## Conclusions

Our research demonstrates that the choice of a data-combining method depends on the characteristics of the data. It is important for researchers to carefully consider the expected magnitude of correlation amongst data sources when estimating disease prevalence using a data-combining method as well as the accuracy of the individual data sources. When correlation between data sources is very high, using the OR method or the AND method will result in comparable estimates of prevalence. When correlation is low, however, we recommend using the OR method. If both data sources tend to poorly capture true non-disease cases, then the AND method is preferable.

In our simulation study, the RSSA method produced large RB and MSE when we underestimated the specificity of case ascertainment algorithms compared to when true estimates of specificity of the case ascertainment algorithms were defined. Therefore, the RSSA method should be used with caution if accurate estimates of sensitivity and specificity of case ascertainment algorithms are not available from published sources. In the simulation, the estimated prevalence from the RSSA method was less biased when true prevalence was 20% compared to 10%. Thus, we recommend using the RSSA when true prevalence is higher, as it is less affected by potentially sparse data.

For the PSSA method, we recommend including a rich set of markers to estimate disease prevalence, especially when true prevalence is low. The PSSA method works best when correlation between the two data sources is high, the average marker correlation is low and the true prevalence is high.

The methods used in this study can be extended to combine more than two data sources. For example, future research could investigate including survey data as a third data source. For example, the population-based Canadian Community Health Survey is used to produce prevalence estimates for many conditions, including hypertension [62], even though it is prone to recall bias. Combining this data source with both AHRs and EMRs might be helpful to epidemiologists and public health staff who routinely use only a single source to report disease prevalence estimates. The PSSA models only included covariates with complete information. However, covariates could potentially be characterized by missing data. Further research could extend this method to account for missingness in the markers [63, 64].

## Additional file

Additional file 1: Visual Graphical Assessment and Trace Plots Showing Convergence for the Probabilistic Sensitivity-Specificity Adjusted (PSSA) Models. Trace plots, density plots and convergence plots of the posterior distribution of the estimated disease prevalence for the PSSA method. (DOCX 2759 kb)

## Data Availability

Data used in this article were derived from administrative health data as a secondary source. The data were provided under specific data sharing agreements only for the approved use. The original source data are not owned by the researchers and as such cannot be provided to a public repository. The original data source and approval for use has been noted in the acknowledgments of the article. Where necessary and with appropriate approvals, source data specific to this article or project may be reviewed with the consent of the original data providers, along with the required privacy and ethical review bodies.

## Abbreviations

AHR: Administrative health record
ATC: Anatomical Therapeutic Chemical
CCS: Charlson comorbidity score
CI: Confidence interval
COPD: Chronic obstructive pulmonary disease
DIC: Deviance Information Criterion
DIN: Drug Identification Number
DPIN: Drug Program Information Network
EMR: Electronic medical record
ICD: International Classification of Diseases
MaPCReN: Manitoba Primary Care Research Network
MCHP: Manitoba Centre for Health Policy
MSE: Mean square error
PHIN: Personal health identification number
PSRF: Potential scale reduction factor
PSSA: Probabilistic-based sensitivity-specificity adjusted
RB: Relative bias
RSSA: Rule-based sensitivity-specificity adjusted
