# Supplementary material for: Combining population-based administrative health records and electronic medical records for disease surveillance
Source: BMC Med Inform Decis Mak. 2019 Jul 2;19:120. doi: 10.1186/s12911-019-0845-5 (PMC6604278; doi:10.1186/s12911-019-0845-5)

**Additional File 1: Visual Graphical Assessment and Trace Plots Showing Convergence for the Probabilistic Sensitivity-Specificity Adjusted (PSSA) Models**

Manuscript Title: Combining Population-Based Administrative Health Records and Electronic Medical Records for Disease Surveillance

Authors: Saeed Al-Azazi, Alexander Singer, Rasheda Rabbani, Lisa M. Lix

Figure S1: Trace plots, density plots and convergence plots of the posterior distribution of the estimated disease prevalence for the PSSA method, overall


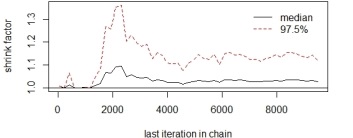

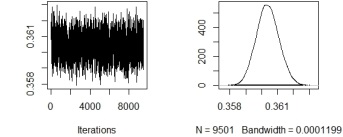

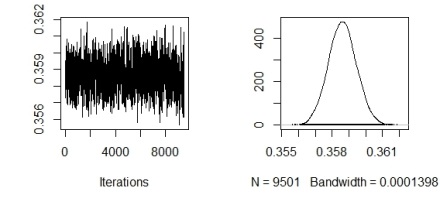

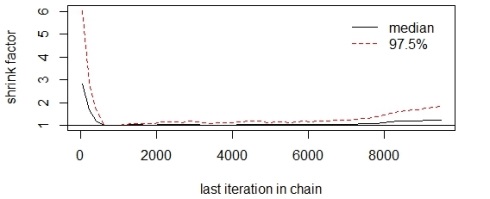

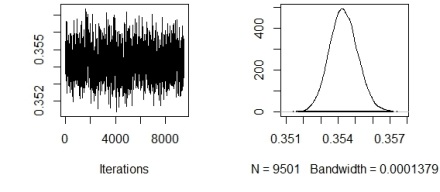

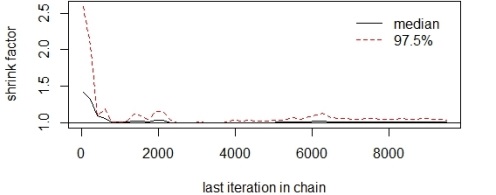

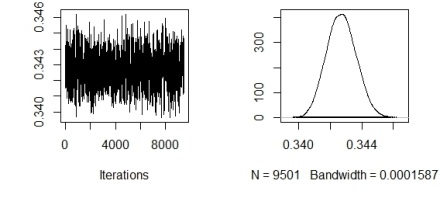

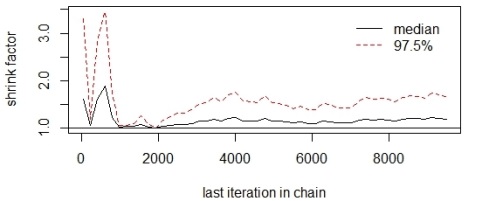


Figure S2: Trace plots, density plots and convergence plots of the posterior distribution of the estimated disease prevalence for the PSSA method, males


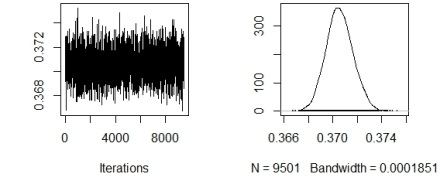

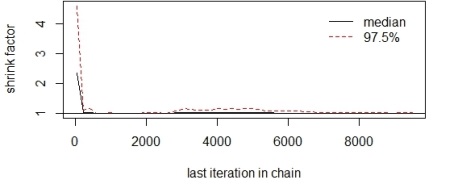

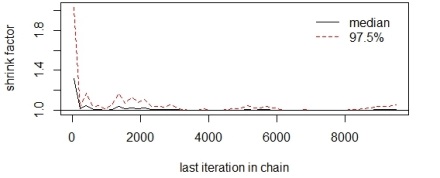

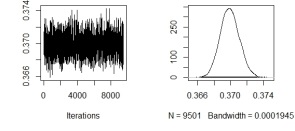

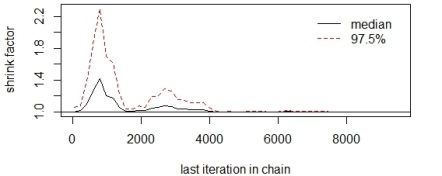

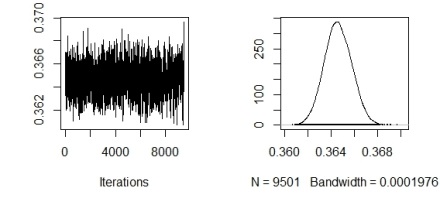

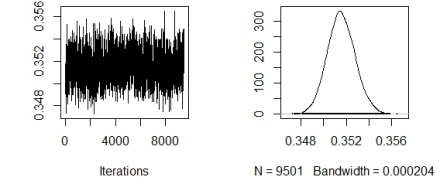

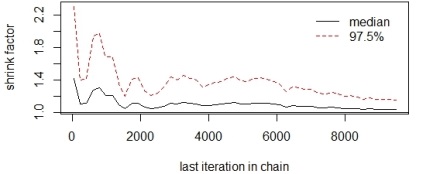


Figure S3: Trace plots, density plots and convergence plots of the posterior distribution of the estimated disease prevalence for the PSSA method, females


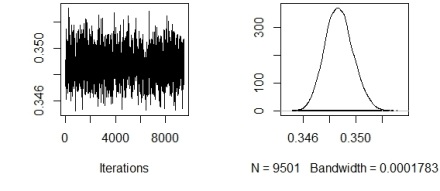

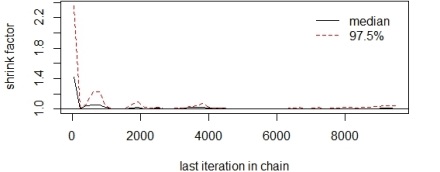

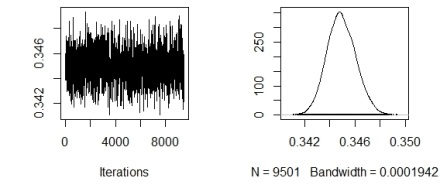

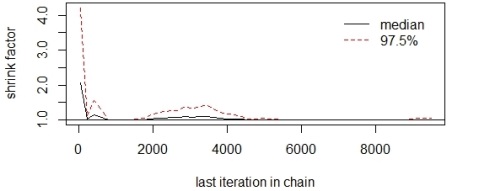

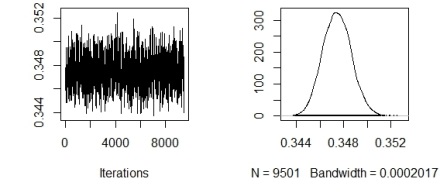

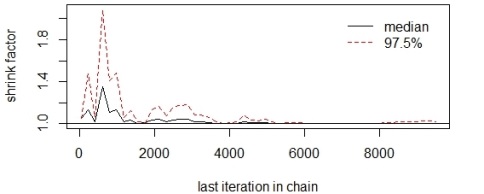

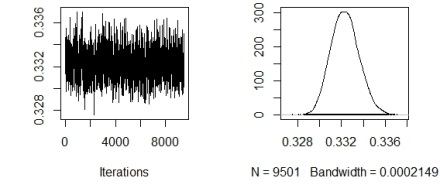

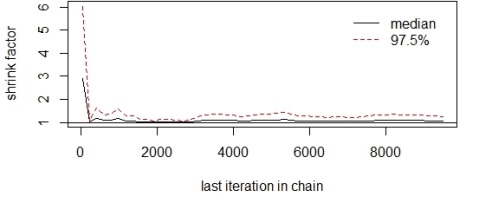


Figure S4: Trace plots, density plots and convergence plots of the posterior distribution of the estimated disease prevalence for the PSSA method, 18-44 age group


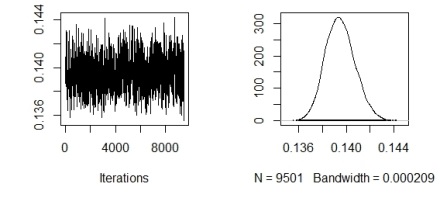

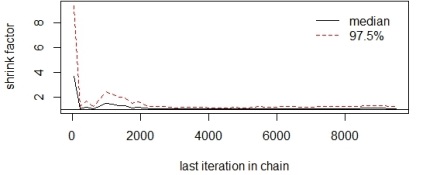

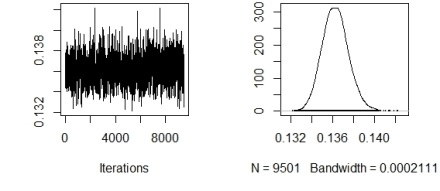

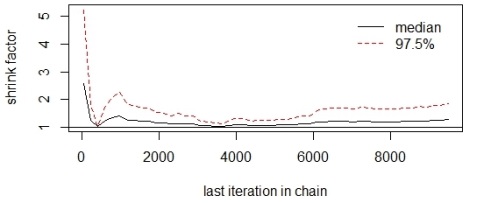

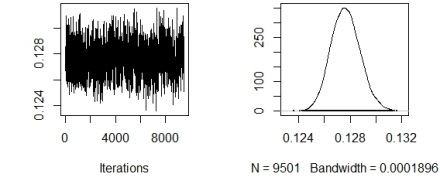

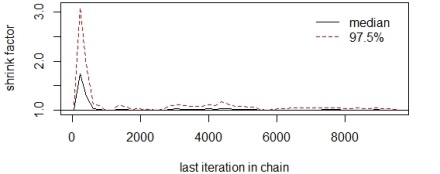

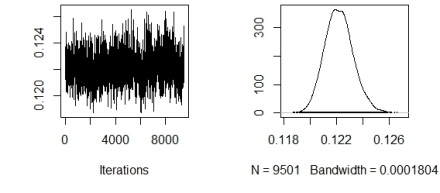

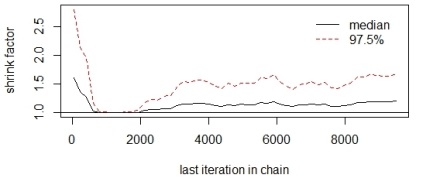


Figure S5: Trace plots, density plots and convergence plots of the posterior distribution of the estimated disease prevalence for the PSSA method, 45-64 age group


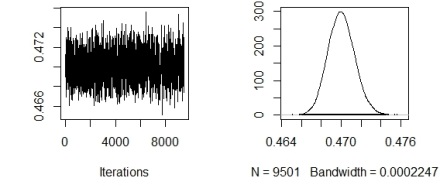

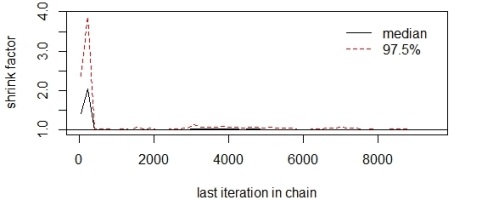

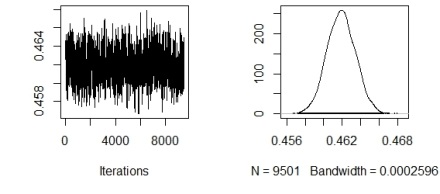

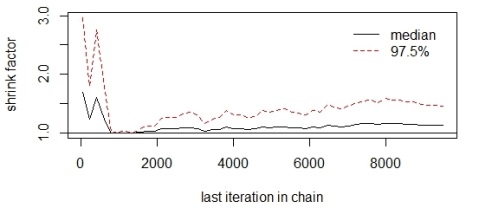

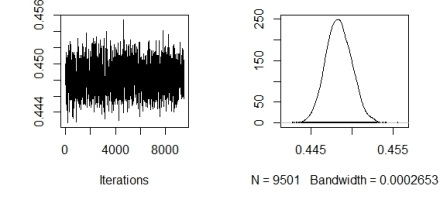

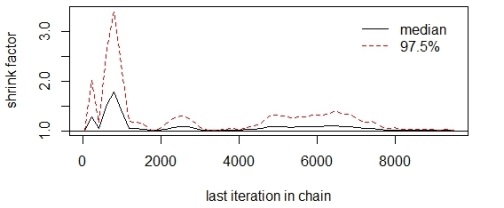

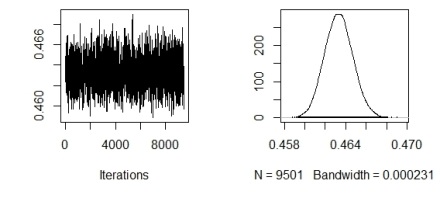

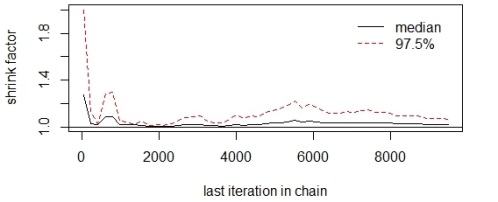


Figure S6: Trace plots, density plots and convergence plots of the posterior distribution of the estimated disease prevalence for the PSSA method, 65+ age group


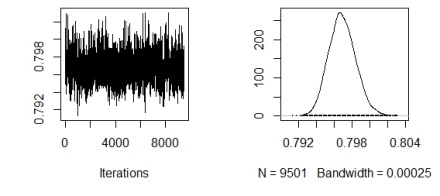

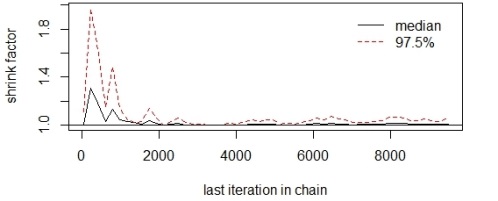

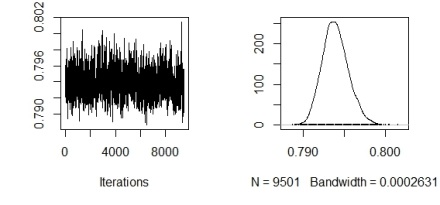

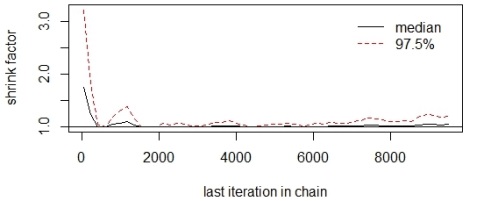

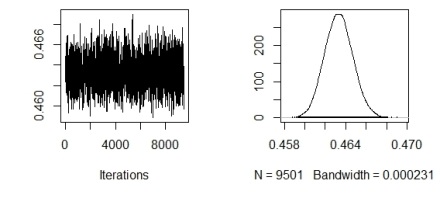

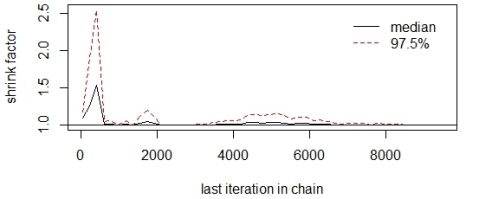

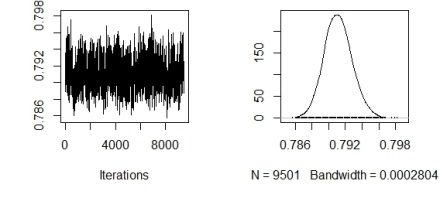

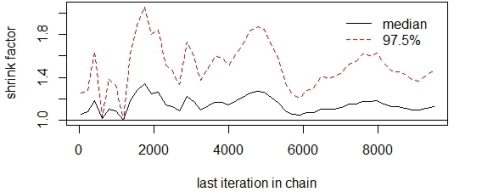

Supplement: Supplementary file 1 — Visual Graphical Assessment and Trace Plots Showing Convergence for the Probabilistic Sensitivity-Specificity Adjusted (PSSA) Models. Trace plots, density plots and convergence plots of the posterior distribution of the estimated disease prevalence for the PSSA method. (DOCX 2759 kb) [file 12911_2019_845_MOESM1_ESM.docx]
